# The Effects of Nicotinamide Adenine Dinucleotide Phosphate (NADPH) Oxidase and Erythropoietin, and Their Interactions in Angiogenesis: Implications in Retinopathy of Prematurity

**DOI:** 10.3390/cells11121951

**Published:** 2022-06-17

**Authors:** Thaonhi Cung, Haibo Wang, M. Elizabeth Hartnett

**Affiliations:** Department of Ophthalmology, John A. Moran Eye Center, University of Utah, 65 Mario Capecchi Dr., Salt Lake City, UT 84132, USA; thaonhi.cung@ttuhsc.edu (T.C.); haibo.wang@hsc.utah.edu (H.W.)

**Keywords:** ROP, angiogenesis, ROS, oxidative stress, NADPH oxidase, NOX, erythropoietin, EPOR

## Abstract

Retinopathy of prematurity (ROP) is a leading cause of vision impairment and blindness in premature infants. Oxidative stress is implicated in its pathophysiology. NADPH oxidase (NOX), a major enzyme responsible for reactive oxygen species (ROS) generation in endothelial cells, has been studied for its involvement in physiologic and pathologic angiogenesis. Erythropoietin (EPO) has gained interest recently due to its tissue protective and angiogenic effects, and it has been shown to act as an antioxidant. In this review, we summarize studies performed over the last five years regarding the role of various NOXs in physiologic and pathologic angiogenesis. We also discuss the effect of EPO in tissue and vasoprotection, and the intersection of EPO and NOX-mediated oxidative stress in angiogenesis and the pathophysiology of ROP.

## 1. Introduction

Retinopathy of prematurity (ROP) is a leading cause of visual impairment and blindness in premature infants. The pathophysiology of ROP has been described by a two-phase hypothesis: Phase 1 ROP is characterized by delayed physiologic retinal vascular development (PRVD) with retinal vaso-attenuation induced by hyperoxia, and Phase 2 ROP is characterized by retinal vasoproliferation into the vitreous, termed extraretinal or intravitreal neovascularization (IVNV) [1]. Severe stages of ROP can lead to vision loss from retinal detachment [2,3].

Oxidative stress has long been implicated in ROP pathophysiology [4,5,6]. The retina and photoreceptors appear vulnerable to reactive oxygen species (ROS) due to their high concentration of polyunsaturated fatty acids [7,8,9]. ROS can activate signaling pathways including those involving apoptosis and angiogenesis that have been proposed in ROP pathogenesis [10]. Nicotinamide adenine dinucleotide phosphate (NADPH) oxidase (NOX) is a major enzyme responsible for ROS generation in endothelial cells [11], and evidence shows that NOX plays a role in ROS-mediated vasculopathies [12]. However, NOX has been shown to be involved in physiologic angiogenesis [12] and in the immune response to invading microorganisms [13], and clinical trials testing antioxidants in ROP have reported inconclusive results [14,15,16]. Recently, erythropoietin (EPO) has gained interest due to its tissue protective and angiogenic effects [17,18], and EPO has also been shown to act as an antioxidant [19,20]. Although multicenter clinical trials and meta-analysis testing exogenous EPO in premature infants did not find evidence of neuroprotection or worsened severity of ROP [21,22,23], another study suggests that EPO is beneficial to the preterm infant without affecting ROP [24]. Other studies, particularly preclinical data, suggest EPO does affect ROP pathophysiology [25,26,27,28,29]. This article will review the role of NOXs in physiologic and pathologic angiogenesis from studies over the past five years and dissect the role of EPO in ROP development and in reducing oxidative stress in angiogenesis.

## 2. Animal Models of OIR

**Figure 1:**
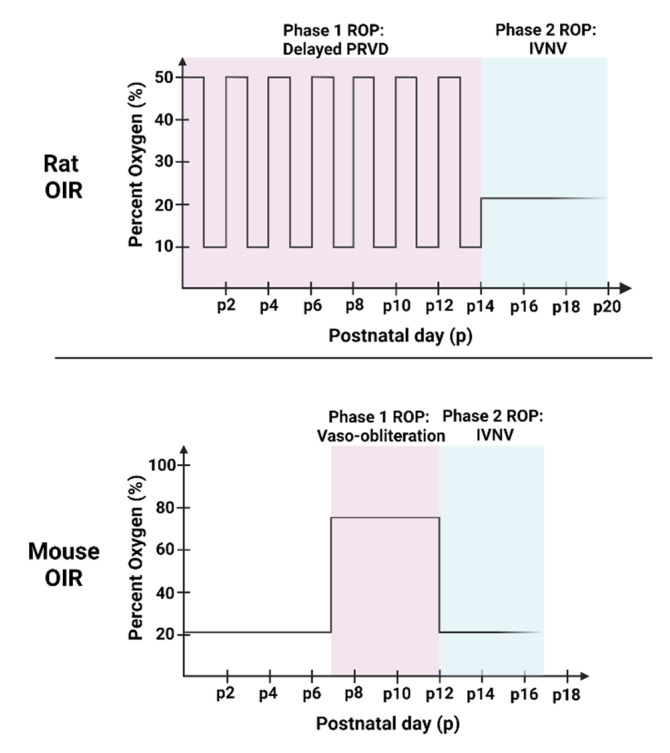
Diagram of the two phases in the rat and mouse OIR models. Phase 1 ROP in the rat OIR is characterized by delayed physiologic retinal vascular development (PRVD) and compromised physiologic vascularity, followed by hypoxia-induced intravitreal neovascularization (IVNV) in Phase 2. Phase 1 in the mouse OIR is characterized by vaso-obliteration followed by hypoxia-induced IVNV in Phase 2. Created with Biorender.com.

Models of oxygen-induced retinopathy (OIR) in mice, rats, beagles, and kittens have been used to study the pathophysiology of ROP [3,30,31,32]. Although the animals in OIR models are not premature, their retinas vascularize after birth, providing an opportunity to study ROP. These models recreate some aspects of pathologic features seen in human ROP, including PRVD and IVNV, and were used to study signaling events implicated in ROP pathogenesis, such as oxidative stress, angiogenesis and inflammation. The rat and mouse OIR models are commonly used to study ROP, and the studies referenced focus on these rodent models. The rat OIR model is the most representative of human ROP because it exposes pups to oxygen fluctuations, causing arterial oxygen levels similar to transcutaneous oxygen levels in human preterm infants at risk of ROP [33,34,35], although the human preterm infant experiences minute-to-minute fluctuations in oxygen [35,36]. Additionally, the oxygen fluctuations cause poor postnatal growth of the pups [31,34,35]. The pups also have retinal vascular features similar to human ROP, including central vaso-attenuation, delayed peripheral PRVD, and extraretinal blood vessels growing into the vitreous (IVNV) [1,2]. In this model, postnatal day 0 (p0) rat pups and dams are placed into an oxygen chamber with oxygen levels fluctuating between 50% and 10% every 24 h for 14 days before being placed into room air (Figure 1). Rat pups have virtually no intraretinal vascularization at p0 and experience delayed PRVD as well as compromised physiologic vascularity at p14 (Phase 1), followed by IVNV, vascular tortuosity, and vascular dilation from p18–p20 (Phase 2) [31]. Another disadvantage to this model is that transgenic rats are not widely available. To study genes in specific retinal cell types, gene therapy with viral vectors has been used to knock down gene expression. Examples include short-hairpin RNAs (shRNA) carried by adeno-associated virus [37], adenovirus [38], and lentivirus [39,40] linked with cell-specific promoters to target cell-specific genes.

**Figure 2:**
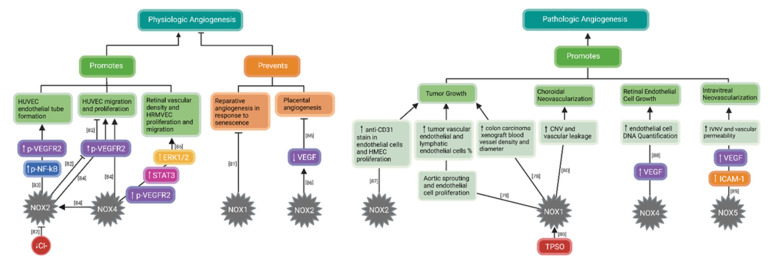
Role of nicotinamide adenine dinucleotide phosphate (NADPH) oxidase (NOX) in physiologic and pathologic angiogenesis. Reduced chloride (Cl^−^) concentration inhibits NOX2 assembly to the active enzyme, resulting in reduced VEGFR2 signaling in HUVEC and decreased cell migration and proliferation [82]. NOX2-generated ROS promote physiologic angiogenesis in HUVEC cells through activation of nuclear factor kappa B (NF-kB) and vascular endothelial growth factor receptor 2 (VEGFR2) [83]. NOX4-generated ROS influence NOX2 activity, and both NOX2 and NOX4-generated ROS increase activation of VEGFR2, resulting in HUVEC migration and proliferation [84]. NOX4 is involved in VEGF-mediated activation of VEGFR2 and downstream signaling of STAT3 and ERK1/2 to promote physiologic vascularization in the retina [85]. NOX1-generated ROS prevent reparative angiogenesis in response to senescence [81], and NOX2-generated ROS downregulate STAT3-dependent VEGF expression and prevent placental angiogenesis [86]. Regarding pathologic angiogenesis, NOX1- and NOX2-generated ROS contribute to tumor growth [78,79,87]. Translocator protein (TPSO) is necessary for NOX1-mediated ROS generation [80], which promotes choroidal neovascularization (CNV) and vascular leakage in a model of age-related macular degeneration [80]. NOX4-generated ROS promote retinal endothelial cell growth in response to oxidative stress [88] and NOX5-generated ROS increase IVNV [89]. Studies are cited in brackets according to their reference number (┴: inhibit; →: promote). Created with Biorender.com.

In comparison, genetically modified mice can be used to study molecular mechanisms when exposed to the OIR model. In the mouse OIR model, murine pups are raised in room air until p7 when inner plexus vascularization has reached the ora serrata [32]. Then, the pups with their mothers are placed into constant 75% O_2_ for 5 days until p12, causing “vaso-obliteration” in the central retina (Phase 1) and are then returned to room air. The relative hypoxia of the avascular retina stimulates angiogenic factors to cause IVNV at the junction between vascular and avascular retina in Phase 2 [32] (Figure 2). The mouse OIR model is not representative of human ROP, because the high oxygen used is avoided in preterm infants now, and the inner retinal vascular plexus is nearly complete when pups are exposed to high oxygen and, therefore, the model does not measure delayed peripheral retinal vascularization [32]. There are other animal OIR models used to investigate the pathophysiologic development of ROP or angiogenesis in response to oxygen stresses, such as the beagle model [30], but not as commonly used.

## 3. NADPH Oxidase (NOX) Involvement in Angiogenesis

### 3.1. ROS Generation from NOX 

*{Note that NADPH oxidase activation through specific subunits will be designated with “NOX” preceding the catalytic subunit number. For example, NOX4 means activated NADPH oxidase with Nox4 subunit.}*

The retina is one of the highest consumers of oxygen in the body [41] and generates abundant ROS [42]. ROS are oxygen-containing ions or radicals that are produced during oxidative metabolism when oxygen accepts free electrons. Commonly recognized radicals include the superoxide radical (O_2_^•-^), the hydroxyl radical (^•^OH), or hydrogen peroxide (H_2_O_2_) [43]. Mitochondria generate ROS through the electron transport chain. In physiologic conditions, by transporting an electron to oxygen, a mitochondrion synthesizes ATP to support cellular metabolism with a relatively small amount of O_2_^•-^ generation. However, pathologic conditions, such as hypoxia and hyperoxia, can lead to dysfunctional mitochondria with reduced ATP production and increased ROS generation from increased membrane permeability, lipid peroxidation, or proinflammatory cytokine production [44,45,46]. Cells have many mechanisms within and outside the mitochondria to neutralize ROS [47]. For example, in the retina, the enzyme mitochondrial superoxide dismutase and the antioxidant glutathione have shown protection against oxidative stress [48,49,50,51].

NOX is the primary source of ROS generation in endothelial cells [11]. Findings from different studies support a notion that NOX-generated ROS regulate both physiologic and pathologic angiogenesis in the retina, depending on Nox subunits expressed and their interactions. There are seven NOXs: NOX1–5, dual oxidase 1 (Duox-1) and Duox-2 [52]. Of these, catalytic subunits, Nox1, Nox2, Nox4, and Nox5, are expressed in vascular endothelial cells [53,54]. All the NOXs except NOX5 require p22phox, a membrane-bound subunit, to be activated and generate ROS [53,55]. NOX1 activation also involves assembly of activated regulatory subunits, NoxA1, NoxO1, p40phox, and Rac1 [55,56]. NOX2 activation requires the assembly of activated regulatory subunits, p47phox, p40phox, and Rac1 [55,57,58]. NOX5 activation does not require cytosolic regulatory subunits because it is activated by intracellular calcium [59]. NOX1, NOX2, and NOX5 can each be activated and individually reduce oxygen to O_2_^•-^, which is eventually converted to H_2_O_2_ by superoxide dismutase [60]. NOX4 is constitutively active due to conjugation with p22phox. O_2_^•-^ generated by NOX4 is rapidly converted to H_2_O_2_, which is not regulated by enzyme-mediated dismutation [61]. Expression of Noxs, the catalytic subunits of NOXs, can be modulated by hypoxia, proinflammatory cytokines such as TNFα, TGFβ, and IFNγ, or by transcriptional and epigenetic mechanisms [62]. Additionally, a number of transcriptional factors are identified to regulate gene expression of *Noxs*, including hypoxia-inducible factor 1 subunit alpha (HIF-1α) [63,64], nuclear factor kappa B (NF-κB) [65], nuclear factor-erythroid factor 2-related factor 2 (Nrf2) [66] and signal transducer and activator of transcription (STAT) [67]. DNA methylation, histone modification [68], and microRNAs [69,70] are also involved in the regulation of *Noxs*. However, mitochondria and NOXs are not isolated systems in generating ROS. More evidence supports crosstalk between mitochondria- and NOX-derived ROS [71]. For example, mitochondria-derived ROS activate NOX to produce more O_2_^•-^, and in turn, NOX-generated ROS increase mitochondrial ROS production. The crosstalk leads to a feed forward loop to amplify intracellular ROS and to disrupt the cellular homeostasis maintained by the balance between ROS and antioxidants, thereby leading to pathology that can affect angiogenesis. The following discussion focuses on further evidence over the last five years and describes dual effects of NOXs in both physiologic and pathologic angiogenesis, including potential relevance to ROP.

### 3.2. NADPH Oxidases (NOXs) in Angiogenesis

The dual effects of ROS in physiologic and pathologic angiogenesis are dependent on the concentration of ROS. At low concentrations, ROS can function as signal transducers to regulate endothelial cell proliferation, migration, and tube formation by promoting angiogenic factor vascular endothelial growth factor (VEGF) expression, VEGF receptor 2 (VEGFR2) signaling, and extracellular-signal-regulated kinase 1/2 (ERK1/2) activation [72]. However, excessive ROS generation can lead to retinal cell damage, particularly photoreceptors, due to the abundance of polyunsaturated fatty acids, which are susceptible to oxidative stress [7,8]. In addition, endothelial dysfunction can occur. Evidence suggests that NOXs play a role in ROS-mediated vasculopathies [73,74]. NOX family members are activated by hypoxia, ischemia, VEGF, angiopoietin, and various growth factors, and they generate ROS that trigger signaling pathways involving angiogenesis [73]. In the rat 50/10 OIR model with supplemental oxygen, pups treated with apocynin to quench retinal ROS effectively reduced IVNV without interfering with ongoing PRVD, suggesting that NOX-generated ROS are involved in IVNV [75]. A later study found that the activation of NOX in the rat OIR model led to IVNV through the Janus kinase 2 (JAK2)/STAT3 signaling pathway [76]. These studies support the role of NOX in pathologic angiogenesis. New findings of NOXs in physiologic and pathologic angiogenesis have dissected the roles of NOX1, NOX2, NOX4, and NOX5 in ocular vascular diseases, cardiovascular diseases, and tumor angiogenesis.

There continues to be conflicting evidence regarding the role of NOX1 in angiogenesis, attributed to other NOX involvement in the different angiogenic phenotypes observed in studies using *Nox1* and other NOX1 subunit knockout animal and cell models [12]. Recent studies reinforce the notion that NOX1-generated ROS promote pathologic angiogenesis [77]. In support of this, knockdown of *Nox1*, the catalytic subunit of NOX1, in HT-29 human colon carcinoma cells diminished tumor growth and blood vessel formation, as measured by blood vessel density and vessel diameter [78]. Another study looked at the effect of NOX1 inhibitor, GKT771, on mice with established colon carcinoma. GKT771 is a novel, highly selective pharmacological inhibitor of NOX1 developed using recombinant cells transfected with Nox1 subunit. GKT771 treated mice with colon carcinoma had reduced tumor growth measured by tumor size and mass and reduced angiogenesis and lymphangiogenesis determined by the percentages of vascular endothelial cells and lymphatic endothelial cells in the tumor mass [79]. Reduced tumor angiogenesis from GKT771 treatment was only observed in *Nox1* sufficient mice, but not *Nox1* deficient mice, suggesting that GKT771 inhibits tumor angiogenesis by targeting NOX1. Additionally, GKT771 also inhibited vascularization in a Matrigel plus assay, aortic sprouting and endothelial cell proliferation [79]. Additional studies in the eye further implicate NOX1 in pathologic angiogenesis. In a murine laser-induced choroidal neovascularization (CNV) model to study age-related macular degeneration (AMD), *Nox1* knockout resulted in significantly reduced laser-induced vascular leakage and CNV volume without altering laser-induced levels of VEGF and angiopoietin. Investigators found that translocator protein (TPSO), a biomarker for reactive gliosis, is necessary for Ca^2+^-associated NOX1-mediated ROS generation in retinal phagocytes, and TPSO knockout resulted in a significant reduction of laser-induced vascular leakage and CNV, further suggesting that NOX1-generated ROS are involved in pathologic angiogenesis in the eye [80]. Additionally, a study involving aging suggests that NOX1-generated ROS prevents reparative angiogenesis as well. Compared to aortas from young mice (3 months), aortas from aged mice (18 months) had three-fold suppressed endothelial sprouting. When aged arteries were treated with NoxA1ds, a putative activation domain of NOX1 activator subunit, NOXA1, the arteries displayed restored endothelial sprouting [81]. Taken together, NOX1-generated ROS promote pathologic angiogenesis and may also inhibit reparative angiogenesis (Figure 2).

NOX4 is the predominant NOX in human retinal endothelial cells (HREC) [90], suggesting its potential effects in retinal angiogenesis. In our previous review, summarized studies suggest that NOX4 mediates vascular recovery after hypoxic and ischemic stress but also promotes pathologic angiogenesis following OIR [12]. In regard to pathologic neovascularization, increased expression of retinal *Nox4*, the catalytic subunit of NOX4, and co-labeling with retinal vessels were found in rat OIR pups at p18 when the maximal amount of IVNV developed [90]. In addition, p17 mice with endothelial specific deletion of *Nox4* developed less IVNV than wild-type mice in OIR, suggesting that NOX4 was involved in IVNV. Nox4 protein was greater in p0 rat retinal lysates from room air raised pups compared to retinal lysates from pups with near complete retinal vascularization at p14 and p18, supporting its role in angiogenesis. An in vitro study suggests that inhibition of NOX4 may be a potential treatment for retinal vasculopathy. HRECs were treated with prolyl hydrolase inhibitor, dimethyloxalylglycine (DMOG), which stabilized HIF activity and led to generated ROS. NOX4 was inhibited with GKT136901 and GKT137831 and resulted in decreased ROS generation, endothelial expression of VEGFA, and endothelial cell DNA quantification, indicating decreased cell growth [88]. Another study provided evidence that NOX4 is involved in retinal vascularization. During OIR, mice with endothelial-specific *Nox4* deletion had reduced vascular density of the superficial, deep, and intermediate layers of the retina at p7, p12, and p17, providing evidence that endothelial NOX4 is important for retinal vascular development in mice [85]. In cultured human retinal microvascular endothelial cells (HRMVECs), knockdown of *Nox4* reduced VEGF-mediated activation of VEGFR2, downstream signaling of STAT3 and ERK1/2, and endothelial proliferation and migration [85,90]. Although endothelial NOX4 activation is implicated in pathologic retinal angiogenesis, it may play a role in physiologic retinal angiogenesis (Figure 2). Therefore, targeting endothelial NOX4 does not appear to be a suitable approach to treat retinal vascular diseases.

There is also conflicting evidence regarding NOX2 involvement in angiogenesis. Previously, ischemic hindlimb models have indicated that NOX2-generated ROS promote physiologic angiogenesis by increasing angiogenic capacity of endothelial progenitor cells (EPCs) [91,92], but hypoxia and ischemia can also activate NOX2 to encourage pathologic angiogenesis [76,93,94]. Recent investigations further elucidate contributors to NOX2 involvement in both physiologic and pathologic angiogenesis through VEGF and VEGFR2 signaling. Deoxyribose-1-phosphate (dRP) is a proangiogenic, endogenous molecule that acts on endothelial cells to promote angiogenesis in an ROS-dependent manner [83]. dRP was shown to intracellularly activate endothelial NOX2 in human umbilical vein endothelial cells (HUVECs), as seen in live cell imaging. NOX2-generated ROS, as measured by dihydroethidium (DHE) fluorescence, activate NF-kB and VEGFR2 to induce endothelial tube formation [83]. Reduced chloride concentration [Cl^−^] was reported to suppress angiogenesis by affecting NOX2 subunit assembly. Reduced [Cl^−^] decreased generation of ROS in HUVECs. Reduced [Cl^−^] also decreased expression of NOX2 catalytic subunits, p22phox and Nox2; translocation of cytosolic regulatory subunits p47phox and p67phox; and p47phox phosphorylation, implicating NOX2 assembly in decreased ROS generation in response to reduced [Cl^−^]. Furthermore, reduced [Cl^-^] prevented oxidative stress-mediated activation of VEGF/VEGFR2 signaling in HUVECs, resulting in decreased HUVEC proliferation, migration, and tube formation [82]. Another study assessing mechanisms of prostate cancer found that NOX2-generated ROS promoted pathologic angiogenesis through VEGF. To model prostate cancer in mice, *Nox2* (the catalytic subunit of NOX2) knockout and wild-type mice were implanted with syngeneic, orthotopic prostate cancer cells. Compared to wild-type mice, *Nox2* knockout mice had reduced angiogenesis in their prostate tumors, as measured by anti-CD31 stained vascular endothelial cells [87]. Investigators previously showed that endosome dysfunction is implicated in prostate cancer [95]. Nox2 subunit was found to co-localize with endosome markers, Appl1, Rab5a, EEA1, and Rab7 in acidified, endothelial endosomes, and in response to VEGFA, NOX2-generated H_2_O_2_ was increased, leading to human microvascular endothelial cell (HMEC) proliferation [87]. However, one study indicates that NOX2-generated ROS prevented physiologic angiogenesis by indirectly inhibiting VEGF. NOX2-generated ROS have been shown to induce apoptosis and have an anti-angiogenic effect [75,96]. Fetal intrauterine growth restriction (IUGR) involves impaired placental angiogenesis. The evidence shows that NOX2 is associated with placental insufficiency, which can occur as a complication in pregnancy, leading to low birth weight infants [86]. Knockdown of *Nox2* subunit with small interfering RNA (siRNA) was found to promote angiogenesis through a mechanism involving upregulation of STAT3-dependent VEGF expression [86]. Altogether, conflicting evidence regarding the role of NOX2 in angiogenesis warrants further studies to decipher the potential of NOX2 as a therapeutic target in vascular diseases (Figure 2).

Interaction between NOXs may also exist, affecting their angiogenic phenotype. In HUVECs, knocking down catalytic subunit, *Nox2* or *Nox4*, by siRNA inhibited VEGF-induced endothelial cell migration and proliferation [84]. Both Nox2 and Nox4 knockdown separately inhibited VEGF-induced VEGFR2 tyrosine phosphorylation, adding to evidence that both isoforms regulate VEGF-mediated VEGFR2 activation and angiogenic effects. However, knockdown of *Nox2* also reduced total VEGFR2 protein, suggesting that NOX2 not only regulated VEGFR2 activation but also regulated VEGFR2 expression. Furthermore, investigators suggested a feed-forward mechanism by which NOX4 interacts with NOX2 to promote angiogenesis. They found that NOX4-generated H_2_O_2_ activated NOX2, as suggested by an increase in mitochondrial O_2_^-^ production, which was shown previously to be inhibited by *Nox2* siRNA. In HUVECs, H_2_O_2_ stimulated increase in pSer36-p66Shc, a mitochondrial ROS regulator, which was also inhibited by *Nox2* siRNA. A previous study showed that pSer36-p66Shc knockdown with siRNA in endothelial cells prevented VEGF-stimulated endothelial cell migration and proliferation [97]. Taken together, these studies suggest that NOX2 can sense NOX4-generated H_2_O_2_ and, thereby, promote mitochondria ROS generation in endothelial cells. Not only does this suggest an interaction between the two NOXs to promote angiogenesis, but it provides a “positive feed-forward ROS-induced ROS release mechanism” for angiogenesis [84] (Figure 2).

NOX5-generated ROS have been found to promote angiogenesis [12,98]. While Nox5 subunit is expressed in human retina, it is not expressed in rodents [99]. However, in a recent study, transgenic mice with inducible human Nox5 expressed in endothelial cell subjected to OIR showed increased IVNV and vascular permeability, as measured by albumin ELISA. Additionally, VEGF and ICAM-1 expression were increased. This study provides evidence that NOX5 is implicated in retinal vasculopathy [89].

In summary, findings from these studies demonstrate that NOXs play important, although conflicting, roles in angiogenesis. NOX1, NOX2, NOX4, and NOX5 are implicated in pathologic angiogenesis. NOX1 may prevent reparative angiogenesis, and NOX2 may prevent physiologic angiogenesis. Evidence supports that NOX2 and NOX4 promote physiologic angiogenesis as well, with possible interactions between them. Targeting a NOX may not be a suitable strategy to treat retinal vascular disease. However, understanding the signaling pathways mediated by NOXs-generated ROS may provide potential therapeutic targets to treat ocular vascular disease.

### 3.3. Intersection between NADPH Oxidase (NOX) and Erythropoietin

Studies involving EPO were initially focused on its hematopoietic potential, but evidence supports its role in other cellular functions, including neuroprotection and angiogenesis. EPO triggers signaling through its classic receptor, EPO receptor (EPOR), which dimerizes and leads to hematopoiesis [100]. EPO binds with high affinity to EPOR. Without EPO binding, EPOR exists as a homodimer. Upon EPO binding, EPOR undergoes a conformational change in which its two intracellular domains are dimerized, causing cross-phosphorylation via pre-bound JAK2. Src-homology 2 (SH2) domain proteins can bind to phosphorylated EPOR and activate transcription factors, including STAT1, STAT3, and STAT5a/b [101]. Dimerized STATs play a role in cell processes including cytoprotection, proliferation, apoptosis, and angiogenesis by regulating the transcription of involved genes. However, EPO can interact with other receptors, such as the β-common receptor or Ephrin receptor B4 [17,102,103], and these interactions have been proposed for other tissue functions including neuroprotection and angiogenesis.

EPO can also exert antioxidant properties and has been tested in the models of optic nerve head injury. Hypoxia upregulated Nox4, the catalytic subunit of NOX4, and EPO expression by hypoxia-inducible factor [63,104]. While some studies indicate that NOX-generated ROS negatively regulate EPO production in response to hypoxia [105,106,107,108], other studies suggest that EPO evokes protection against NOX-mediated oxidative stress from hypoxia and ischemia. For example, EPO treatment reduced expression of Nox2 subunit n rat models [109,110]. After exposure to intermittent hypoxia, intraperitoneal administration of EPO to rats significantly reduced the cortical levels of p47phox starting at 3 days and sustained thereafter, correlating with improved spatial learning performance [111]. Another rat model looked at the effect of EPO in mitigating stress in the form of renal ischemia–reperfusion injury (RIRI). To model ischemia (i.e., RIRI) in rats, blood flow to the left renal pedicle was obstructed with a vascular clamp for 30 min, and the right kidney was removed to induce ischemia to the left kidney. In response to RIRI, intraperitoneal administration of EPO reduced *Nox4*, *p22phox* mRNA, and ROS generation in the kidney by a mechanism involving adenosine phosphate monophosphate kinase (AMPK) activation [112]. EPO was also found to protect against vascular and endothelial dysfunction from NOX-mediated oxidative stress. Using a nitric oxide synthase inhibitor to recreate hypertension in a rat hypertension model, administration of oral recombinant human EPO reduced NOX-dependent O_2_^•-^ production, enhanced the expression of suppressor cytokine signaling-1, and improved vasodilation [113]. In a streptozotocin-induced diabetic rat model, subcutaneous administration of human recombinant EPO quenched NOX-generated superoxide in the aorta and improved aortic vessel relaxation in response to acetylcholine [114]. However, some studies also support that NOX-generated ROS facilitate EPO signaling, particularly in vascular repair. Endothelial progenitor cells (EPCs) are known to contribute to vascular repair, and mobilization of EPCs into the sites of vascular injury is an essential step for vascular repair and new blood vessel formation [115]. EPO is a potent stimulator of EPC mobilization [116], and *Nox2* mRNA is highly expressed in EPCs [117]. In *Nox2*-deficient mice, EPO-induced EPC mobilization into an injured carotid artery was not observed, and transplantation of bone marrow from *Nox2*-deficient mice into wild-type mice also interfered with EPC mobilization in response to EPO, supporting the premise that NOX2-generated ROS are important for EPO-induced EPC mobilization. Additionally, EPCs transfected with *Nox2*-sense oligonucleotides showed significantly reduced EPO-mediated STAT5 transcription. These findings provide evidence that NOX2-generated ROS facilitate EPO signaling in promoting hypoxia-induced EPC mobilization and vascular repair [117].

In summary, these studies suggest interactions between NOX and EPO and that EPO might attenuate NOX-induced pathology in diseases such as ROP. In the following section, we discuss the role of EPO in the pathophysiology of ROP.

## 4. Erythropoietin Involvement in Angiogenesis

### 4.1. EPO-Mediated Protection against Vaso-Obliteration and Delayed Physiologic Retinal Vascular Development

Human infants with stage 4 ROP had significantly higher levels of EPO in the vitreous compared to infants with congenital cataracts [118]. In the mouse OIR model, EPO mRNA levels were lower during the 5 days in 75% oxygen or immediately after compared to mice raised in room air [25,26], suggesting that lack of EPO contributes to initial vascular loss in the mouse OIR model. Therefore, the investigators tested if exogenous EPO prevented oxygen-mediated vascular loss and IVNV using the mouse OIR model. Treatment with systemic EPO reduced hyperoxia-induced retinal vascular loss in a time-dependent and dose-dependent manner. Early intraperitoneal injection of EPO at p6 and p7 significantly reduced avascular area compared to later injection when mice returned to room air. Additionally, increased doses of early intraperitoneal EPO injection exhibited a dose-dependent protective effect on avascular retina [25]. Suppressed expression of *Epo* mRNA by intravitreal injection of *Epo* siRNA at later time points, p12 and p14, reduced vaso-obliteration seen in p17 retina in the mouse OIR model; however, intravitreal injection of *Epo* siRNA at p15 resulted in no significant difference in vaso-obliteration in p17 retina compared to control negative siRNA IV injection [26]. The investigators suggested that the effect of EPO could be both beneficial and detrimental depending on the timing in the mouse OIR model. In the rat OIR model, early intraperitoneal EPO injection into pups at p2, p4, and p6 resulted in significantly improved PRVD in p14 compared to PBS injected pups [27]. These studies suggest that timing and dosage of exogenous EPO intervention is important. Additionally, another study in a rat model of uteroplacental insufficiency (UPI) with combination of the OIR model found that rat pups born to moms with UPI had reduced avascular retinal area at p18 compared to pups born to control moms, suggesting an improved PRVD. The study connected the increased PRVD in UPI pups with an increase in serum EPO [119].

Studies have looked at the role of EPOR signaling in angiogenesis. Suzuki et al. developed mice that only expressed EPOR in cells from erythroid lineage and lacked EPOR expression in nonhematopoietic tissue (*EPOR^−/−^* rescued mice) [120]. Therefore, *EPOR^−/−^* rescued mice expressed EPOR in hematopoietic but not in other tissue. With these mice, investigators showed that EPOR contributes to VEGF expression and promotes angiogenesis in response to hind limb ischemia. Two weeks after femoral artery ligation, activation of the VEGF/VEGFR system and mobilization of EPCs were impaired in *EPOR^−/−^* rescued mice compared to wild-type mice, suggesting that EPO/EPOR regulates ischemia-induced angiogenesis through VEGF/VEGFR signaling [121]. To delineate the effect of EPO signaling through EPOR, humanized knock-in *EPOR* mice with hypoactive EPOR signaling were compared to littermate wild-type mice. Hypoactive EPOR signaling showed reduced retinal vascular coverage before p7 when the inner plexus vascularization to the ora serrata is usually completed [32]. After exposure to hyperoxia in the mouse OIR model, hypoactive EPOR signaling also resulted in increased central avascular area. These findings suggest that EPO signaling through EPOR is implicated in PRVD before oxygen insult, and that EPOR signaling protects against hyperoxia-induced vascular loss [29].

### 4.2. EPO-Mediated Increase in Hypoxia-Induced Pathologic Neovascularization

Evidence provides support that EPO extends PRVD, but its angiogenic potential might exacerbate IVNV [25,26]. Retinal EPO mRNA expression was increased during hypoxia in the mouse OIR model following hyperoxia-induced avascularity [25,26]. Intravitreal *Epo* siRNA given following hyperoxia reduced IVNV compared to control [26]. In addition, treatment with EPO or EPO mimetics increases HRMVEC proliferation [122], the number of EPCs in the retinas of p8 mice exposed to oxygen [25], and endothelial colony-forming cell tubulogenesis [123]. In addition, intraperitoneal injections of EPO delivered early at p6, p8, p10, and p12 in the mouse OIR model reduced IVNV in p17 retina, but late intraperitoneal injections of EPO after hyperoxia did not reduce IVNV compared to control injected in p17 retina [25]. Therefore, protection from EPO treatment may depend on timing. HIF-α and HIF-1α-like factor (HLF) are both important in embryonic vascularization and upregulation of angiogenic factors [104,124], including EPO and VEGF. *Hlf* knockout mice with corresponding partially reduced endogenous EPO expression and wild-type mice were placed into the mouse OIR model; some *Hlf* knockout mice were also treated with intraperitoneal EPO during relative hypoxia. Compared to wild-type mice, *Hlf* knockout mice had significantly reduced retinal neovascular buds, suggesting reduced IVNV. Moreover, EPO treatment of p12 *Hlf* knockout mice had increased retinal neovascular budding measured at p17 compared to control treated *Hlf* knockout mice [125]. Altogether, these studies support the notion that EPO levels relate to pathologic IVNV in animal OIR models, although further studies need to be performed to elucidate the effect of timing.

**Figure 3:**
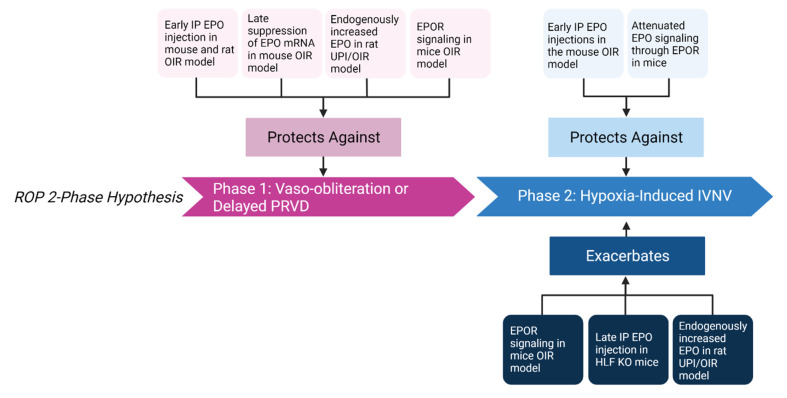
Effect of EPO and EPOR signaling on Phase 1 and Phase 2 ROP. Early treatment indicates that treatment was given prior to or at the start of Phase 1 ROP or hyperoxia-induced vascular loss; late treatment indicates that treatment was given at the end of or after Phase 1. Studies show that early exogenous intraperitoneal (IP) erythropoietin (EPO), late EPO suppression, endogenously increased EPO from uteroplacental insufficiency (UPI), and erythropoietin receptor (EPOR signaling) protect against vaso-obliteration or delayed PRVD in oxygen-induced retinopathy (OIR) animal models. Early exogenous EPO and attenuated EPO signaling through EPOR protects against Phase 2 hypoxia-induced intravitreal neovascularization (IVNV); however, late EPO injection in HIF-1α-like factor (HLF) knock out (KO) mice endogenously increased EPO from uteroplacental insufficiency (UPI), and EPOR signaling has been shown to exacerbate IVNV in animal OIR models. Created with Biorender.com.

Studies examining the role of EPOR signaling in the development of IVNV suggest that EPOR contributes to IVNV. EPOR expression and activation were increased in retinal vascular endothelium in the rat OIR model compared to room air raised rat pups at p14 and p18 [126]. One group of investigators used soluble recombinant EPOR from Chinese hamster ovary cells [127], which when administered, binds with higher affinity to EPO, attenuating EPO signaling through EPOR [128]. They reported that intravitreal injections of the soluble recombinant EPOR in mice raised in OIR at p12 and p14 significantly reduced intravitreal neovascular nuclei in a dose-dependent manner compared to volume-control injections of human IgG [128]. A different group used humanized mice in which the murine *EpoR* gene was replaced with the human *EPOR* gene, causing hypoactive signaling through EPOR due to a downstream transmembrane protein and not ligand-receptor binding [29]. Littermate wild-type mice with sufficient EPOR signaling had increased vascular growth in mice from p3 to p7 compared to mice with hypoactive EPOR signaling, suggesting that signaling through EPOR promotes physiologic retinal vascularization. In OIR, littermate wild-type mice had reduced avascular area at p17 but did not show a difference in IVNV compared to mice with hypoactive EPOR signaling. This suggests that signaling through EPOR also contributes to hypoxia-induced regrowth after OIR [29]. In addition, littermate wild-type mice with sufficient EPOR signaling also experienced neuroprotection following OIR with reduced thinning of retinal layers and improved electroretinographic function [28]. The data suggest that EPOR signaling can support physiologic retinal vascularization, can be vasoprotective in high oxygen and neuroprotective in development and after OIR, and can support regrowth following oxygen-induced damage and IVNV (Figure 3). Future studies are considered to delineate the role of EPOR and alternative EPO receptors in the pathophysiology of ROP.

## 5. Conclusions

ROP is the leading cause of visual impairment and blindness in premature infants. Oxidative stress has been thought to play a role in the development of ROP, and NOX is implicated in ROS-mediated vasculopathies. However, NOXs may not be appropriate targets for treatment because of their involvement in immune response to invading microorganisms and their role in both physiologic and pathologic angiogenesis. Studies from the last five years support the evidence that NOX1, NOX2, NOX4, and NOX5 are implicated in pathologic angiogenesis. Additionally, NOX1 and NOX2 may prevent reparative and physiologic angiogenesis, respectively. However, NOX2 and NOX4 are also involved in physiologic angiogenesis with possible interactions between them. EPO has been considered for its tissue-protective and angiogenic properties. There is evidence that EPO can act as an antioxidant and can interact with NOX. The studies summarized suggest that EPO protects against NOXs-generated ROS-mediated endothelial dysfunction and vascular damage. In animal models of OIR, early exogenous EPO appears to protect against hyperoxia-induced vascular loss, but late exogenous EPO can contribute to pathologic IVNV. Additional studies are warranted to examine the role of EPO signaling through EPOR as well as alternative receptors and the potential interaction with NOX-generated ROS.
